# The Repertoire of Heterotrimeric G Proteins and RGS Proteins in *Ciona intestinalis*

**DOI:** 10.1371/journal.pone.0007349

**Published:** 2009-10-06

**Authors:** R. Prasobh, Narayanan Manoj

**Affiliations:** Department of Biotechnology, Indian Institute of Technology Madras, Chennai, India; Max Planck Institute for Evolutionary Anthropology, Germany

## Abstract

**Background:**

Heterotrimeric G proteins and regulators of G protein signaling (RGS) proteins are key downstream interacting partners in the G protein coupled receptor (GPCR) signaling pathway. The highly versatile GPCR transmembrane signaling system is a consequence of the coupling of a diverse set of receptors to downstream partners that include multiple subforms of G proteins and regulatory proteins including RGS proteins, among others. While the GPCR repertoire of *Ciona intestinalis*, representing the basal chordate is known, the repertoire of the heterotrimeric G proteins and RGS proteins is unknown.

**Methodology/Principal Findings:**

In the present study, we performed an *in-silico* genome-wide search of *C. intestinalis* for its complement of G proteins and RGS proteins. The identification of several one-to-one orthologs of human G proteins at the levels of families, subfamilies and types and of homologs of the human RGS proteins suggests an evolutionarily conserved structure function relationship of the GPCR signaling mechanism in the chordates.

**Conclusions:**

The *C. intestinalis* genome encodes a highly conserved, albeit, limited repertoire of the heterotrimeric G protein complexes with the size of subunit types comparable with that in lower eukaryotes.

## Introduction

G protein coupled receptors (GPCRs) comprise a large family of diverse transmembrane signaling proteins that receive information from various extracellular stimuli including hormones, neurotransmitters or sensory stimuli. The three-component GPCR signaling system is so named based on its ability to recruit and regulate the activity of intracellular heterotrimeric G proteins (guanine nucleotide-binding protein). Ligand binding at the extracellular recognition sites on the GPCRs is transduced into intracellular signals through the coupling of the receptor and G protein and between G protein and other effector proteins, all of which can be independently regulated by additional proteins or at the transcriptional level.

The heterotrimeric G protein is composed of α, β, and γ subunits. The inactive form of the GDP-bound heterotrimeric state is activated when the agonist activated receptor induces a conformational change in the G protein trimer resulting in the Gα-subunit binding to GTP in exchange for GDP. This exchange leads to the dissociation of Gα-GTP and Gβγ subunits that further interact with downstream target effectors thus activating and regulating a signaling cascade. The turnoff of the cellular response occurs when the Gα subunit hydrolyses GTP to GDP and the Gα-GDP and Gβγ subunits re-associate to form the Gαβγ trimer. The Gβ and Gγ subunits work as an obligate complex, a functional unit that can only be dissociated under denaturing conditions. The structural and functional aspects of G proteins and their receptor-mediated activation have been extensively studied [1]–[5]. The third component of the GPCR signaling system is the G protein-regulated effector. Several effectors enhance GTPase activity of the Gα subunit thus playing a role in deactivation and modulation of G protein mediated signaling. A recent modification to the standard model of GPCR signaling has come from a family of proteins called “regulators of G protein signaling” or RGS proteins that have been found to accelerate the intrinsic GTPase activity of Gα subunits independent of the Gβγ subunits [6].

The modular architecture of the G protein-mediated transmembrane signaling system is highly versatile and specific and is based on the fact that there are numerous receptors (around 800 in human) and several types of G proteins and effector proteins [7]. Enormous diversity of heterotrimeric complexes can be assembled from a limited repertoire of G protein subunits which are then activated by different receptors [8]. Most receptors also are able to activate more than one type of G protein [9]. Moreover, several Gβγ complexes can interact with the same Gα suggesting that differential expression or subcellular localization are important in the regulation of downstream signaling [10].

*Ciona intestinalis* (henceforth referred to as *Ciona*) is a protochordate belonging to the ascidian class of chordates that diverged from the vertebrate lineage about 520 million years ago. An out-group to the vertebrates, this ascidian has the smallest genome of any experimentally manipulable chordate. A translucent morphology, availability of developmental mutants, quickly spawning embryos, established transgenic, morpholino-based gene knockdown, *in situ* hybridization experimental procedures and extensive EST data are some of the many advantages that make *Ciona* an excellent model organism to study developmental and evolutionary biology of the vertebrate-invertebrate split. Moreover, *Ciona* possesses organ systems that are homologous to vertebrate heart, thyroid, blood, digestive and neural complex systems [11]–[14]. In an earlier study, a genome wide survey of the repertoire of GPCRs in *Ciona* reported the presence of 169 putative receptors. A comparative analysis of the repertoire revealed a high level of orthology with that of human GPCRs (about 40% of *Ciona* receptors) [15]. Here we extend the previous study and report the identification of the repertoire of the heterotrimeric G proteins and the RGS proteins in *Ciona* and present a comparative analysis with that in human. The analyses could provide insights into the origin and evolution of the GPCR signaling system in a protochordate and its further diversification into the vertebrate lineage. Our results thus could serve as a basis for carrying out experimental studies to address functional and regulatory aspects of GPCR mediated signaling.

## Materials and Methods

### Protein data mining

The Joint Genome Institute (JGI) *C. intestinalis* genome versions 2.0 and 1.0 databases (http://genome.jgi-psf.org/Cioin2/Cioin2.home.html; http://genome.jgi-psf.org/ciona4/ciona4.home.html) were used as the source for obtaining the complete proteome [16]–[17]. The v2.0 proteome dataset includes 15,852 proteins and sequences in the current version include both automated and to a lesser extent, manually curated annotations. The sequences of the query G proteins and RGS proteins were obtained from the NCBI non-redundant database and the gpDB Database [18]. The gpDB Database is a relational database of GPCRs and GPCR interacting proteins based on interactions reported in the literature. The BLAST program was used for initial identification using a cut off of E-value of 10^−3^ [19]. Additionally, searches were carried out using customized Hidden Markov Models (HMM) built separately for all known families/sub-families listed in the gpDB Database. The HMMs were constructed using HMMBUILD and HMMCALIBRATE programs of HMMER package version 2.3.2 [20]. *Ciona* sequences that returned hits with an E-value better than 0.01 were extracted. Default settings were used in all HMMBUILD and HMMCALIBRATE models constructed. At this stage of the analysis, E-values of low stringency were used for BLAST and HMM search algorithms to enable detection of all true positives. False positives, if any, were sifted out in the next stage with a higher level of stringency where the results of BLAST, best reciprocal BLAST hit and HMM based search were compared and common hits satisfying the cutoff criteria were taken as confirmed hits of *Ciona*. All except two of *Ciona* sequence accession numbers reported in this study, including that of several manually edited fragments, refer to the identifiers assigned in the JGI v2.0 genome database. GenBank accession numbers, where available, are given in the supporting information.

### Sequence comparison and phylogenetic methods

Multiple sequence alignment of the *Ciona* sequences with known orthologs picked up from the gpDB database was performed using CLUSTALX 1.8 program [21] using the default parameters for substitution matrices and gap penalties. Manual adjustments of the alignments were made when necessary using the BioEdit program [22]. All phylogenetic analysis was carried out using the PHYLIP package, as implemented in the MOBYLE Portal (http://mobyle.pasteur.fr/) [23]. Neighbor-Joining (NJ) and Maximum-Likelihood (ML) methods as implemented in the NEIGHBOR and PROML programs were employed for tree searching and inference. The statistical reliability of the phylogenetic trees was tested by interior branch analysis with 500 bootstrap replicates used to create a consensus tree. Phylogenetic trees created using the NJ method and the ML method resulted in trees with identical topologies and clusters with significant bootstrap support. Alignments and secondary structures were displayed using ESPript 2.2 (http://espript.ibcp.fr) [24].

### Expressed Sequence Tag hits and tissue/developmental stage based expression data

The entire UniGene database for the *Ciona* transcript sequences was downloaded from the ftp site (ftp://ftp.ncbi.nih.gov/repository/UniGene/) and locally installed. All *Ciona* G protein and RGS protein sequences were queried against the UniGene EST database using TBLASTN with the E-value set at 1e^−5^. Identifiers of the statistically significant EST-hit collection were imported in an excel sheet and categorized based on their derivative developmental stage/tissues.

## Results and Discussion

In order to generate an independent data set of the repertoire of G proteins and RGS proteins, a data mining approach was taken using BLAST program and HMM based searches. Sequences from the gpDB Database and the NCBI were collected and classified into different groups and families of G proteins and RGS proteins. The sequences were carefully chosen to represent diverse organismal lineages and groups as reported in the gpDB classification. The search strategy that included using sequences as single queries and also using distantly homologous sequences combined into a profile for a more sensitive HMM based search, resulted in the identification of unambiguous homologs in the *Ciona* proteome. The query sequences used for the BLAST searches and for the construction of HMMs are available as supplemental data (Data S1). Further classification of the *Ciona* sequences into orthologous sets of different classes, families, subfamilies and types were carried out based on phylogenetic analyses (Table 1, Table S1). The complete amino acid sequences of all identified *Ciona* proteins are available as supplemental data (Data S2).

**Table 1:** Classification of heterotrimeric G proteins and RGS proteins in *C. intestinalis*.

Class	Family	Subfamily	Type	JGI Accession *C. intestinalis* sequence^a^
Gα	G_12/13_	Gα _12,_ Gα _13,_ Unclassified G_12/13_	Gα _12_	287420 (Q14344–59%)
	G_i/o_	Gα gust	Gα gust	-
		Gα_i_	Gα_i,_ Gα_i1,_ Gα_i2,_ Gα_i3_	209567 (P63096–83%)
		Gα_o_	Gα_o_, Gα_io1_	-
		Gα_t_	Gα_t_, Gα_t1_, Gα_t2_	-
		Gα_z_	Gα_z_	-
	G_q/11_	Gα_11_, Gα_14_, Gα _15/16_, Gα_q_, Unclassified G_q/11_	Gα_11_, Gα_14_, Gα _15/16_, Gα_q_, Unclassified G_q/11_	272522 (P50148–74%), 390373 (P29992–42%)
	G_s_	Gα_olf_	Gα_olf_	-
		Gα_s_	Gα_s_	208025 (P63092–63%)
	Unclassified Gα	Unclassified Gα	Unclassified Gα	281048 (P63096–33%)
	Other Gα	Other Gα	Other Gα	220350 (P63096–59%) 273922 (P20353–56%) 202119 (P63096–61%) 287065 (P63096–59%)
Gβ	Gβ	Gβ	Gβ_1_, Gβ_2_, Gβ_3_, Gβ_4_	283613 (P62873–78%)
			Gβ_5_	297548 (O14775–57%)
	Unclassified Gβ	Unclassified Gβ	Unclassified Gβ	-
Gγ	Gγ	Gγ	Gγ (1–5,7,8, 10–13), Gγ_e_, Gγ_t1_, Unclassified Gγ	225022 (O60262–40%) ci0100144696 (Q9UBI6–35%)
RGS with GGL domain	R7	R7	RGS6,RGS7,RGS9, RGS11	373767 (P49802–55%)

a data in parenthesis indicates the UNIPROT accession and values of sequence identity with the top human sequence match.

Since EST evidence covers three quarters of the predicted *Ciona* genes, we also identified EST matches for the predicted *Ciona* G proteins and RGS proteins. Except for 2 out of 10 Gα sequences and 5 out of 14 RGS sequences, all other sequences had at least one EST match mapped to the gene sequences. EST matches for these sequences were sorted as being derived from different developmental stages as well as tissues (Table S2).

### The Gα subunit

The Gα class of the heterotrimeric G proteins comprises four main families, Gα_s_, Gα_i/o_, Gα_q/11_ and Gα_12/13_, each consisting of several members with different expression patterns. Members of these families are structurally conserved and often share functional properties. The gpDB Database lists four other families that include members from nematodes, fruitfly, fungal_plant and one group designated as ‘Unclassified Gα”. The human repertoire of Gα class as listed in the gpDB database includes a total of 31 sequences with each of the families Gα_s_, Gα_i/o_, Gα_q/11_ and Gα_12/13_, containing 9, 13, 5 and 2 members, respectively. In our study, a total of 10 unambiguous orthologs of the Gα class were identified in *Ciona*. Three sequences out of the ten members were fragments, possibly the result of errors in the automated splice site prediction methods used. In these cases, additional searches carried out against the NCBI non-redundant protein database and the *Ciona* proteome v1.0, yielded two sequences that were full length proteins. The validity of the edited sequences was verified by comparison of these regions against the EST database. In the case of the third fragment (281048), the missing region was identified by the use of translated alignments against the *Ciona* genomic regions where the missing region was expected to be found based on alignment with the human orthologous protein. However, in this case, the EST database contained no hits corresponding to the genomic region. The *Ciona* Gα homologs share a relatively high pair wise sequence identity ranging from 33–83% with the best match in human (Table 1). The sequence identity values within the *Ciona* Gα proteins range from 26–99%.

**Figure 1:**
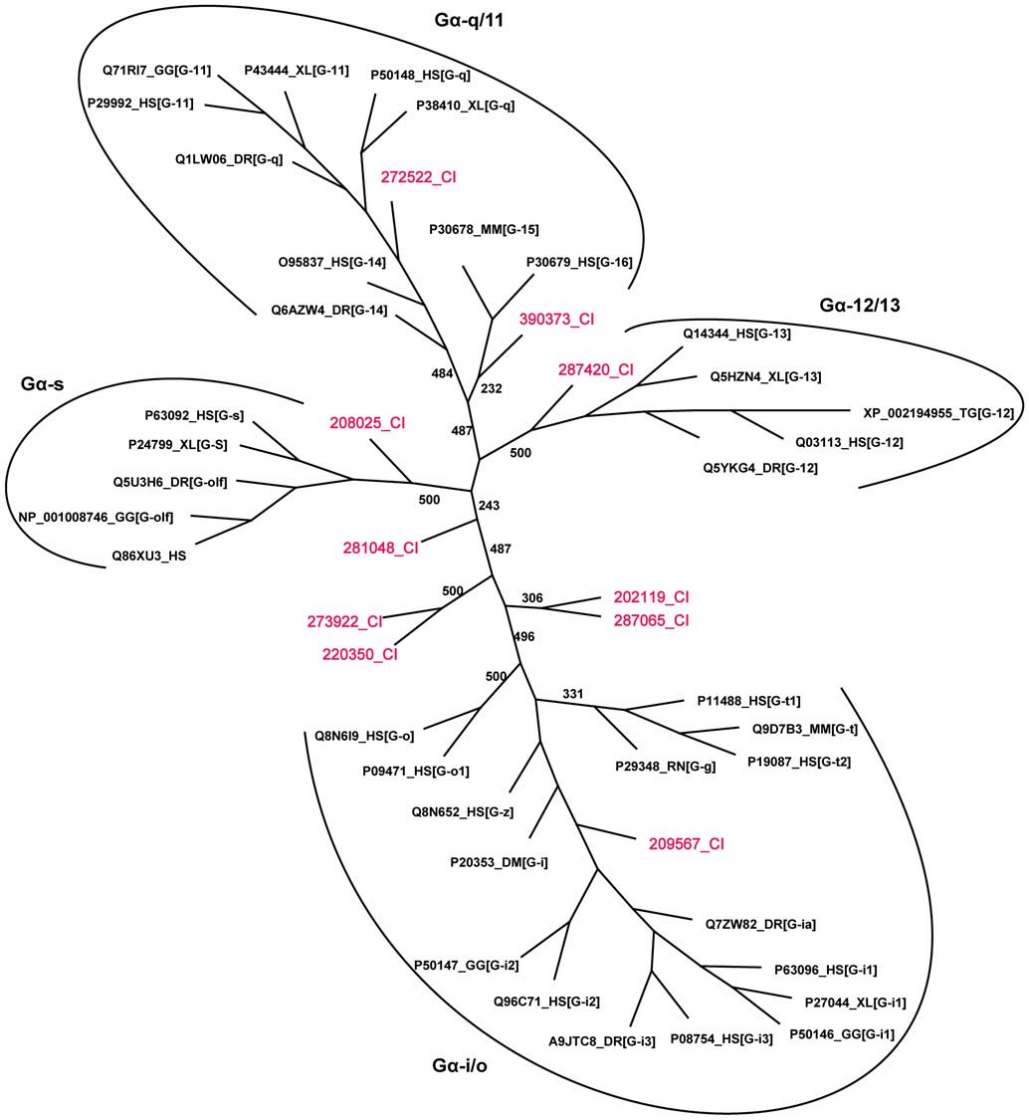
Phylogenetic relationship between Gα subunits in *Ciona* and other genomes. The figure illustrates the unrooted tree of Gα subunits in *Ciona* and representative organisms. Clusters that represent the four main families of Gα proteins are marked. Bootstrap support values (out of 500 data sets) are shown. Abbreviations used in this figure and figures 2,3,4,5a and 5b are HS- human, DM- *Drosophila melanogaster*, MM- mouse, RN- rat, CI- *Ciona*, CE- *C. elegans*, DR- *Danio rerio*, XL- *Xenopus laevis*, TG- *Taeniopygia guttata*, GG- chicken. Family names are given in square brackets.

**Figure 2:**
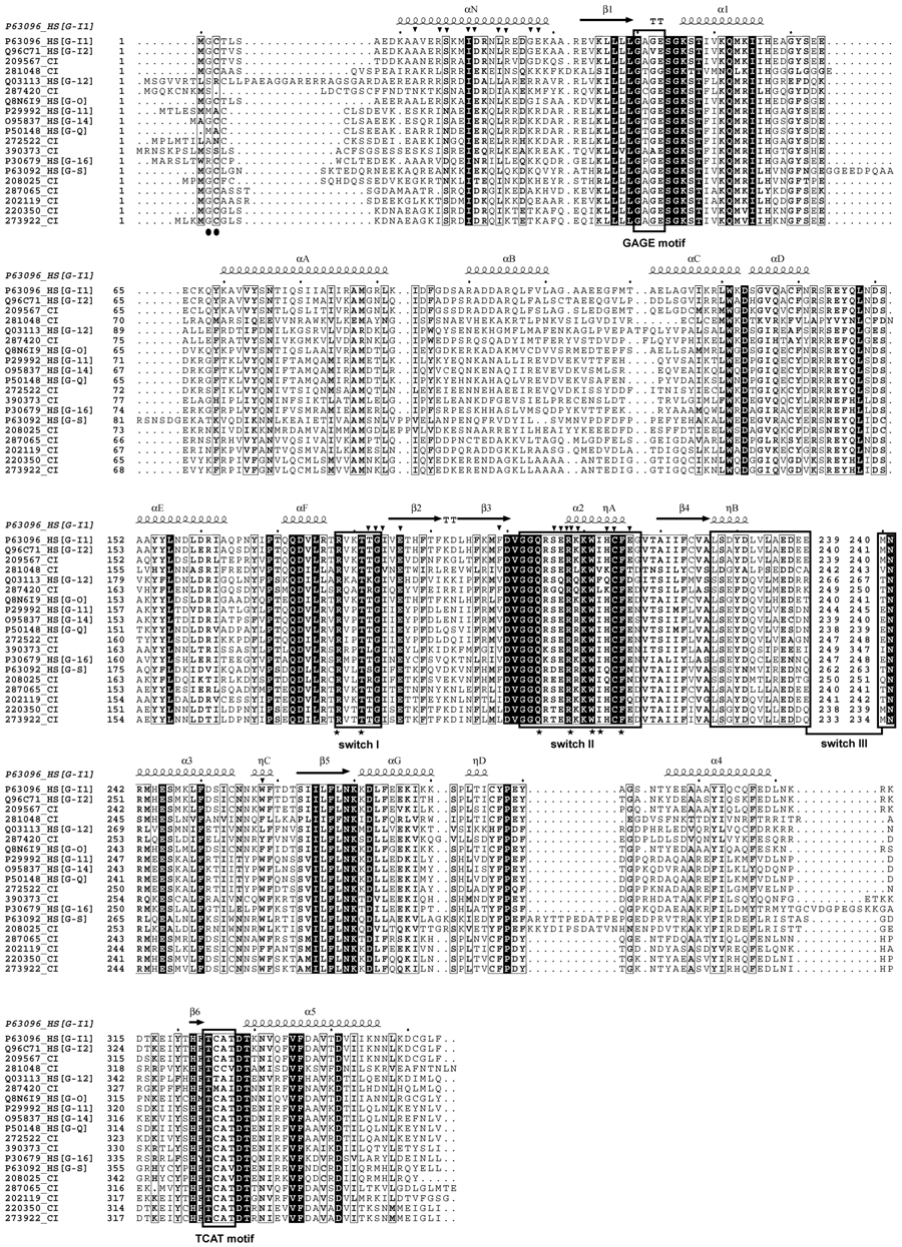
Multiple sequence alignment and secondary structure features of the Gα subunit. Secondary structure assignments are derived from mammalian Gα_i1_ (PDB ID: 1GP2). The three conformationally flexible switch regions of Gα (including the entire α2 helix within switch II) are indicated in rectangular boxes; residues in Gα that contact Gβ are marked with inverted triangles above the ruler line. Conserved Gα guanine base and phosphate contact positions are indicated with rectangular boxes and are marked correspondingly (GAGE, DVGGQ motifs and TCAT motif); the conserved Arg, Thr and Glu residues involved in GTP hydrolysis and key switch II residues (Lys209, Trp211, Ile212, and Phe215) that interact with Gβ_1_ are represented by stars.

The classification of the *Ciona* sequences into different families and subfamilies, carried out by phylogenetic analysis, revealed distinct clusters that corresponded to known families and subfamilies in human and other organisms (Figure 1). The phylogenetic tree showed monophyletic clades with good bootstrap support for each of the major families. The clustering shows two orthologs of the G_q/11_ family and one ortholog each of the Gα_i/o_ family, the Gα_s_ family and the Gα_12/13_ family. The Gα_i/o_ ortholog (209567) shows the highest identity to that of human. We however, did not find *Ciona* orthologs of the three non-vertebrate families. Five *Ciona* homologs clustered into three separate groups with no clear one-to-one orthologous relationship to the known families. However, among these, two *Ciona* pairs (220350/273922; 201119/287065) which are located basal to the main Gα_i/o_ family grouping, share high sequence identities of about 59% to the human Gα_i1_. One of these pairs, (220350/273922) shares an identity of 99% and appears to be a recent gene duplication event. The two genes have the same loci and are positioned in tandem to each other, separated by about 3500 nucleotides. These divergent *Ciona* specific Gα proteins were placed into a group designated ‘Other’. One *Ciona* Gα sequence (281048) was found to be an outgroup to all the other clusters. Although this homolog retains all the critical and conserved motifs, it shows slight deviations in two consensus motifs, namely the GAGE motif at the N-terminal and the TCAT motif at the C-terminal regions of the sequence (Figure 2). This homolog also is the most divergent among the *Ciona* Gα proteins (26–34%) and shares an identity of 33% with the top human Gα match. Hence this homolog was placed into a group designated ‘Unclassified’. The presence of only a single or two members in *Ciona*, belonging to each of the major Gα subunit families is in contrast to the presence of several members of each family in mammals. Moreover, a lower eukaryote like *Caenorhabditis elegans* has 20 Gα subunit genes out of which four belong to the four major mammalian families, while the remaining 16 are not homologous to the mammalian subunit and are a product of lineage specific gene expansion [25].

The Gα subunits in *Ciona* share a strong level of sequence conservation with those from other organisms including human, reflecting common structure-function relationships (Figure 2). The Gα subunit contains two domains, a Ras-like domain with a nucleotide-binding pocket and an all α-helical domain, composed of a six-helix bundle that makes up a lid on the nucleotide-binding pocket. Binding to GTP causes conformational changes within three flexible segments of Gα, namely “switch” regions (I–III) leading to a well ordered, GTP bound activated conformation with lowered affinity for the Gβγ complex and increased affinity for downstream effectors. Several crystal structures of Gα-effector complexes have revealed that a common site for effector interactions is provided by a highly conserved hydrophobic cleft within active Gα, formed by the α2 and α3 helices. The region of sequences making up these helices are highly conserved between *Ciona* and human sequences, implying similar mechanisms of Gαβγ complex/Gα-effector recognition and binding. Interestingly, one *Ciona* homolog (390373), displays an unusual insertion of about 150 amino acids in the switch III loop that connects the α2 and α3 helices. This functional loop is ordered only in the active state of Gα. The triad of Gα residues that mediates GTP hydrolysis includes Thr181 and Arg178 in switch I that stabilize the γ-phosphate ion and Gln204 in switch II which coordinates the critical nucleophilic water molecule responsible for hydrolysis of the γ-phosphate, is completely conserved (numbered as in human Gα_i1_, Figure 2) [26].

The communication between activated GPCR and the Gα subunit is modulated primarily through the C terminus/α5 helix and α4/β6 loop that invoke further structural changes necessary for GDP release [27]–[29]. The loop connecting β6/α5 contains a completely conserved TCAT motif that stabilizes the binding of GDP. The release of GDP is triggered by the receptor contacts to the C terminus and through the α5 helix that further structurally modulate the TCAT motif. Other highly conserved regions of the Gα subunit involved in transmitting conformational changes of the TCAT motif includes the α3 helix, which connects the α3/β5 loop to switch III and the β6 strand [30]–[31], [5]. The N-terminal region of the *Ciona* Gα subunits also contains highly conserved Cys and Gly residues that are sites of palmitoylation and myristoylation which anchor the subunits to the membrane thus regulating membrane localization and protein-protein interaction [32].

### The Gβγ complex

**Figure 3:**
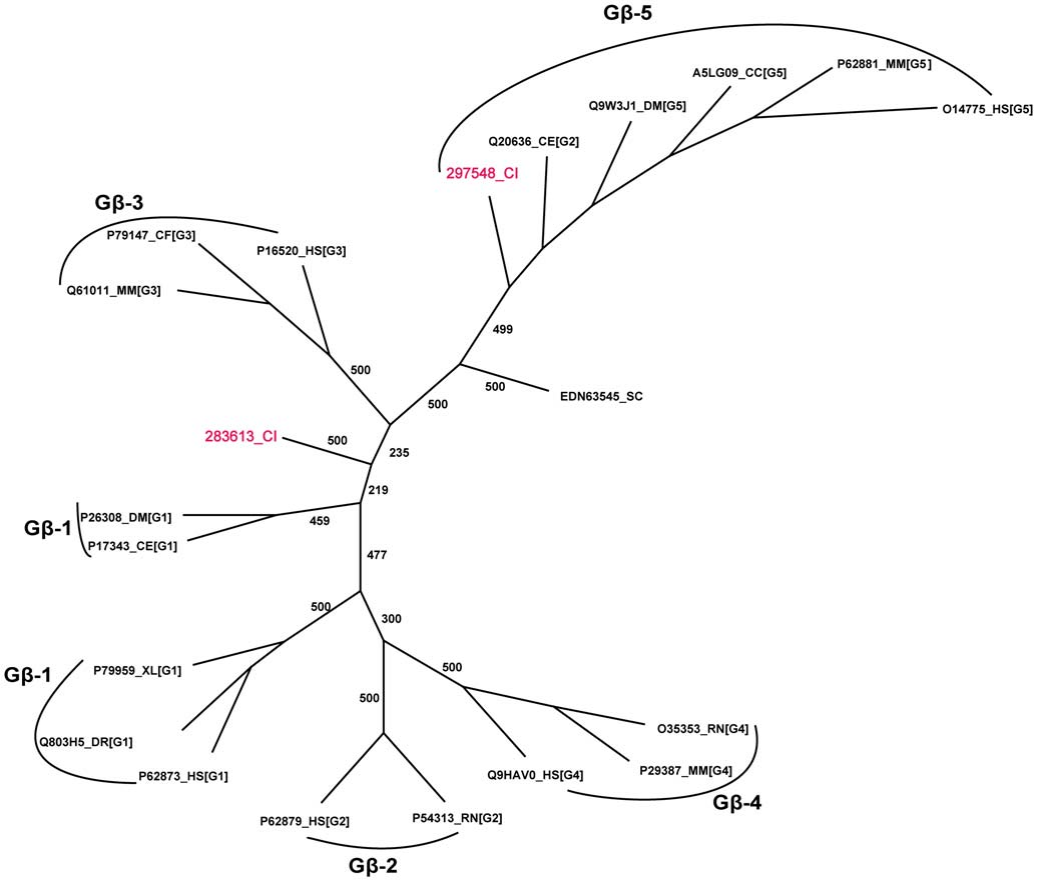
Phylogenetic relationship between Gβ subunits in *Ciona* and other genomes. The tree illustrates the unrooted tree of the Gβ subunits in *Ciona* and representative organisms. The five major clusters represent the five types of Gβ subfamilies. *Ciona* has only two types, in that, one is an ortholog of the Gβ_5_ type while the other, an ortholog of the Gβ(1–4) type group, is equidistant to each of the four types. Bootstrap support values (out of 500 data sets) are shown. Abbreviations used are CC- *Cyprinus carpio*, CF- *Canis familiaris*, SC- for *Saccharomyces cerevisiae*. Gβ type names are given in square brackets.

A total of two homologs of the Gβ class were identified in *Ciona* with both homologs exhibiting the characteristic conserved set of WD repeat motifs. Figure 3 denotes the relationships of the repertoire of *Ciona* Gβ homologs. The Gβ class of proteins consists of a single family and subfamily that includes five types of Gβ proteins named Gβ(1–5). In the mammalian genome, the types 1 to 4 are highly conserved sharing greater than 80% sequence identity, but Gβ_5_ is divergent, sharing only about 50% identity [33]–[34]. Our phylogenetic analysis indicates that one *Ciona* homolog (283613) exhibits a clear one-to-one orthologous relationship to the Gβ(1–4) group while the other sequence is orthologous to the Gβ_5_ members (297548) (Figure 3). The two *Ciona* Gβ members share a sequence identity of about 41% between them and are more divergent than that in human. The *Ciona* Gβ(1–4) ortholog shares a high sequence identity of about 77% with that of the human Gβ(1–4) group while sharing about 40% identity with the human Gβ_5_. The *Ciona* Gβ_5_ ortholog shares a sequence identity of 51% with the human Gβ_5_.

**Figure 4:**
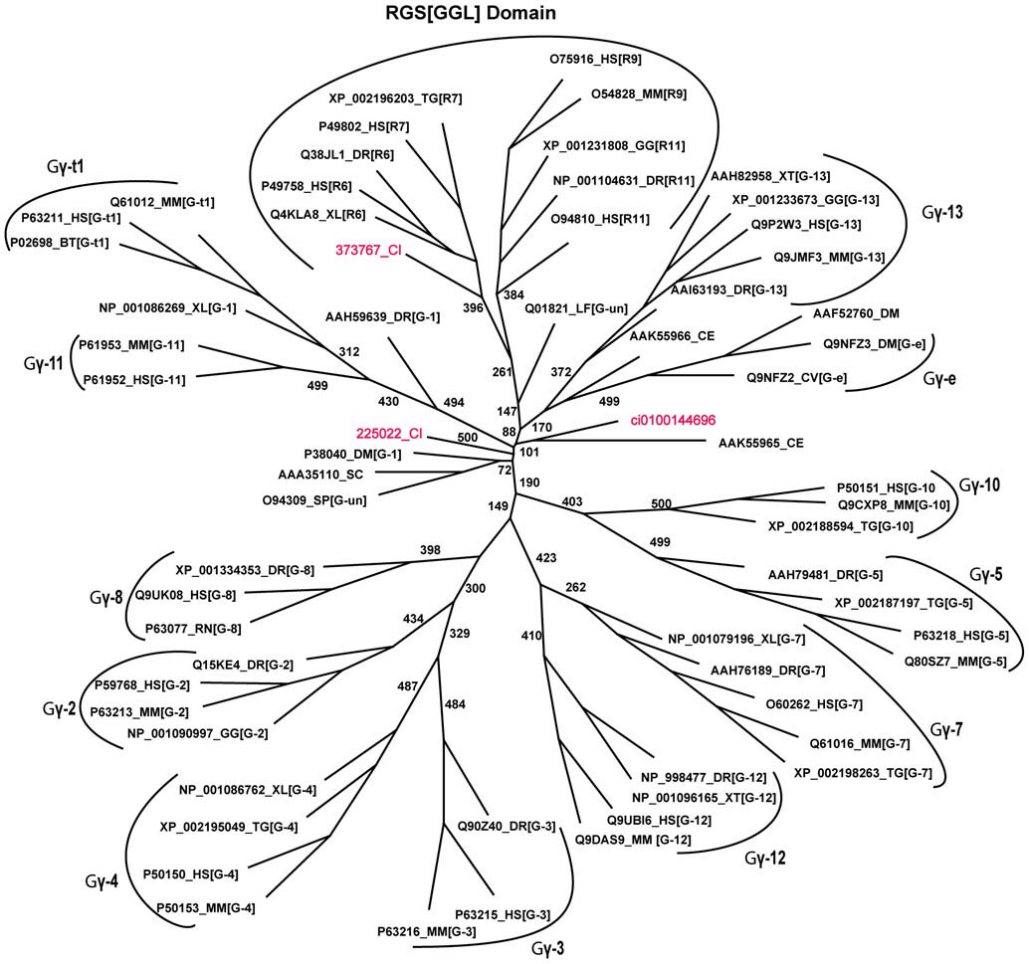
Phylogenetic relationship between Gγ subunit of *Ciona* and other genomes. The figure illustrates an unrooted tree of the Gγ subunits in *Ciona* and representative organisms. Different clusters represent the different types of Gγ subfamilies. The GGL domains form a separate significant cluster. The single Gγ subunit in *Ciona* is an outgroup to all the other members. Abbreviations used are LF- *Loligo forbesi*, SP- *Schizosaccharomyces pombe*, CV- *Calliphora vicina*, XT*- Xenopus tropicalis*. Gγ type names are given in square brackets. Un- refers to ‘Unclassified’ sequence.

The *Ciona* genome holds two homologs of the Gγ class of proteins. It has to be noted here that a search for Gγ homologs in *Ciona* v.2.0 database resulted in the identification of just a single hit. However, parallel searches of the v1.0 database resulted in the identification of yet another homolog of the Gγ protein that has not been modeled and annotated in the v2.0 database. We have therefore annotated this sequence by the accession number corresponding to the *Ciona* v1.0 genome database. In both Gγ homologs, the protein models have been verified as having EST hits. In human, 12 Gγ gene products are known. Furthermore, among all heterotrimeric G protein subunits identified in *Ciona*, the Gγ homologs display the largest divergence to that of human. The *Ciona* Gγ sequences share the highest sequence identities of 40% and 34% to human Gγ type 7 and 12 members. Phylogenetic analysis of the Gγ members revealed that the *Ciona* Gγ subunits show no reliable clustering to any known Gγ types as classified in the gpDB database (Figure 4).

**Figure 5:**
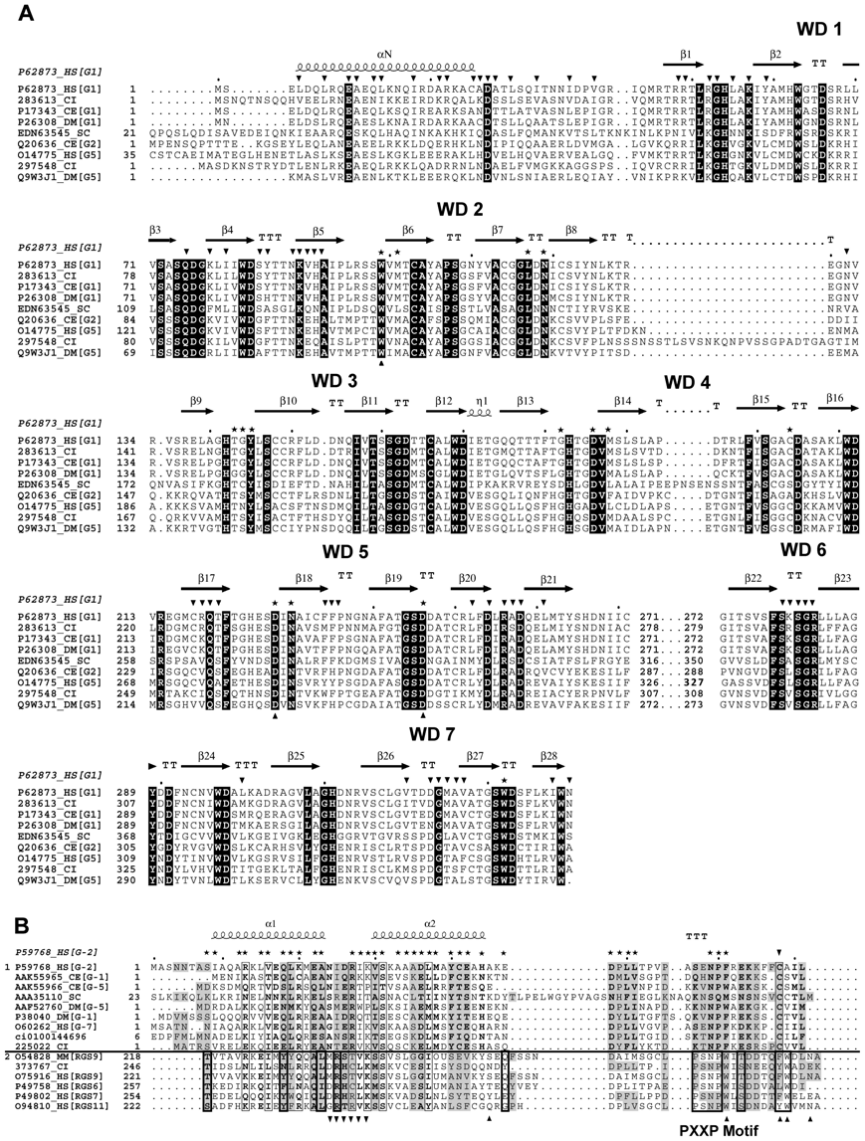
Multiple sequence alignments and secondary structure features of Gβ subunits, Gγ subunits and GGL domains. A. Alignment of the Gβ sequences from representative organisms. Secondary structure assignments (α-helices, β-strands) are derived from mammalian Gβ_1_ (PDB ID: 1GP2). The four β-strands that comprise each of the seven WD repeat segments within Gβ subunits are marked with horizontal arrows to match the tertiary structure of Gβ_1_. Residues in Gβ that contact Gγ are marked with inverted triangles and the contacts with the switch regions in Gα are indicated with stars. Key Gβ_1_ residues (Trp99, Asp228, and Asp246) interacting with the Gα_i1_ switch II helix are represented with upright triangles. B. Alignments of the Gγ subunits and GGL domains from representative organisms. Secondary structure assignments are derived from mammalian Gγ_2_ (PDB ID: 1GP2). Gγ subunit–like (GGL) domains and Gγ subunits are structurally equivalent. Residues in Gγ that contact Gβ are marked with stars. Bold letters indicate highly conserved residues. The inverted triangle in the C-terminal of Gγ sequences represents prenylation site. The horizontal line in the alignment separates the two blocks of Gγ and the RGS sequences (GGL domain region). The black vertical boxes in the lower block show conserved residues specific to the GGL sequences. The inverted triangles corresponds to residues in the RGS9 of human and mouse that make contacts with Gα subunit, while the upright triangles corresponds to the specific contacts with the Gβ_5_ subunit.

The Gβ subunit has an extended N terminal α-helix followed by a seven-bladed β-propeller fold that is composed of seven WD sequence repeats. The N-terminus is primarily supported by coiled-coil interactions with Gγ which is an extended stretch of two α-helices joined by a loop with additional interactions to loops that connect the CD and AB strands of Gβ blades 5 and 6 [35]–[36] (Figures 5a,b). The heterotrimeric Gα and Gβγ complex formation involves two major sites of interactions. Firstly, the extensive burial of the β3/α2 loop and α2 helix (switch II) of Gα within six of the seven WD repeats of Gβ. Numerous interactions involve a hydrophobic core formed by Trp211 of Gα and Trp99 of Gβ (numbering as in human Gβ_1_) and forms the basis for Gβγ-mediated guanine nucleotide dissociation inhibitor activity and for Gβγ binding between Gα-GDP and Gβγ-effectors [5]. Several key switch II residues such as Lys209, Trp211, Ile212 and Phe215 that interact with Gβ_1_ are completely conserved in *Ciona*. The other major region of Gα/Gβ interactions involves the WD1-2 of Gβ and the extended N-terminal helix of Gα (Figure 2, Figures 5a,b). There is also considerable overlap of the effector binding site on Gβγ with the region that interacts with the switch II of Gα [37]. Our sequence comparisons reveal that a majority of the functionally important residues of the Gβγ sequences are identical between *Ciona* and other organisms including human, indicating highly conserved modes of complex formation.

The selectivity and diversity of the Gβγ complex of mammalian G proteins arise from the numerous potential dimer combinations that can be assembled from a repertoire of 5 Gβ protein subunits and 12 Gγ subunits. The Gβ(1–4) subunits form a tight complex with Gγ subunits and can only be separated under denaturing conditions [38]. The sequence divergence between the Gβ(1–4) and the Gβ_5_ types is reflected in the relatively weak interactions of the Gβ_5_ with the Gγ subunits in contrast to that in the Gβ(1–4)γ complex. Moreover, unlike the other Gβ subunits which are generally found associated with the membrane, Gβ_5_ exists in a significant amount in the soluble cell fraction [39]. It is now well established that Gβ_5_ makes an *in-vivo* complex with the R7 subfamily of the RGS proteins in preference to Gγ and that the novel Gβ_5_-RGS dimer formation is primarily mediated through interactions via a Gγ-like domain (GGL) present N-terminal to the RGS domain [34], [40]. The identification of the Gβ_5_ ortholog in *Ciona* also points to the fact that the *Ciona* genome most likely encodes homologs of GGL domain containing RGS proteins.

### RGS proteins

**Figure 6:**
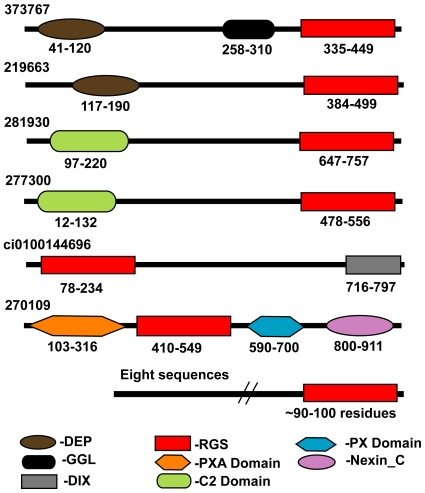
Domain organization of the repertoire of RGS proteins in *Ciona*. Eight *Ciona* sequences have just the RGS domain with varying lengths of the N-terminal region, while six sequences have other types of domains as well. DEP (Dishevelled, Egl-10, and Pleckstrin domain); RGS (Regulator of G protein signaling domain); GGL (Gγ like domain), Nexin_C (Sorting nexin C terminal domain); PX (Phox domain).

The repertoire of human RGS family includes at least 37 members that are broadly classified into eight subfamilies. All members contain a core Gα-interacting RGS domain while they differ widely in their over all sizes and organization of several functional domains. RGS proteins are multifunctional and have the ability to negatively regulate Gα subunits and also function as effector antagonists. This multiple modular architecture enables regulatory selectivity and specificity in their mediation with diverse signaling partners that include all three components of the GPCR signaling pathway [41], [6]. We identified 14 proteins in *Ciona* that possess the RGS domain (Table S1, Data S1). Eight of these are sequences that contain just the RGS domain at the C-terminal end with N-terminal regions having variable lengths ranging from 28 to 349 amino acid residues. It is to be noted here that 9 sequences identified from *Ciona* v2.0 database turned out to be fragments and in all these cases, the complete sequences corresponding to these members were identified in v1.0 database. One other fragment sequence (ci0100153349) was exclusively identified only from the v1.0 database with no corresponding protein model in v2.0. The complete sequence corresponding to this member was identified in the GenBank database. An examination of the RGS protein sequences for additional domains carried out using the Conserved Domain Database (CDD) indicated that six sequences contain additional domains that could confer further functionality [42]. One member has a DEP (Dishevelled, Egl-10 and Pleckstrin) domain N-terminal to the RGS domain, while two sequences contain a C2 domain in addition to the RGS domain. The RGS repertoire also includes one sequence that contains four domains that include an N-terminal PXA (Phox-associated) domain, an RGS domain followed by a PX (Phox) domain and a Nexin_C domain and another sequence with an RGS domain and a C-terminal DIX domain (Figure 6). Among these various domains, the DEP domain has been shown to play an important role in mediating the interaction of an RGS protein to the C-terminal tail of the GPCR, thus placing RGS in close proximity with its substrate Gα subunit [43]. The PX domain is an important phosphoinositide-binding module with varying lipid-binding specificities and implicated in cell signaling.

Our results further revealed that *Ciona* indeed does contain one RGS homolog (373767) that contains a GGL domain located between the DEP and RGS domains analogous to the domain organization that characterizes members of the R7 subfamily (RGS6, RGS7, RGS9, RGS11) of the human RGS family (Figure 6). This finding strongly supports the formation of an RGS complex with the Gβ_5_ ortholog in *Ciona*. Sequence comparison of the GGL domain with that of the Gγ subunits from *Ciona* as well as other organisms indicates the homologous relationship of the GGL domain to the Gγ subunit (Figure 5b). This is further confirmed by the existence of significant monophyletic clades that separates the Gγ and GGL domains (Figure 4). The GGL domain of *Ciona* shares an identity of 17% and 19% with the Gγ subunit in *Ciona* and human, respectively and shares an identity of 28–49% with the four GGL domains in human. Recent structural studies show that the marked difference in specificities of Gβ_5_ for the GGL domain versus Gγ arise from the cumulative effect of several minor changes in the corresponding sequences [44]. It is evident from our comparative analysis that the *Ciona* GGL domain containing RGS homolog could indeed interact with the Gβ_5_ homolog in an analogous mode.

### Heterotrimeric G protein complexes

The results of our analysis indicating a repertoire of only one homolog each of the Gβ(1–4) type and the Gβ_5_ type subunits, two homologs of Gγ subunit and one homolog of the GGL containing RGS protein implies that only a maximum of five potential Gβ heterodimer complex combinations (two Gβ(1–4) γ complexes, two Gβ_5_γ complexes and one Gβ_5_-RGS complex) likely exists in *Ciona*. Furthermore, with 10 Gα subunits, the *Ciona* genome potentially displays a limited repertoire of 50 Gαβγ heterotrimeric complexes. It is noteworthy that the diversity in the types of Gβγ complexes in *Ciona* is comparable to those seen in lower eukaryotic model organisms. For example, yeast has one Gβ subunit (Genbank ID: AAA35114) that is equally distant to the mammalian Gβ1-4 and the Gβ_5_ types. *C. elegans* has two Gβ subunit sequences that are each orthologous to the Gβ1-4 and the Gβ_5_ types. Furthermore, yeast has a single Gγ subunit (Genbank ID: AAA35110) that shows poor similarity to the human homolog, while *C. elegans* has two Gγ subunits (Genbank ID: AAK55965, AAK55966) that do not fall into any particular mammalian subfamilies (Figure 4).

### Conclusions

*Ciona* appears to have a highly compact set of the heterotrimeric G protein complexes and these results complement the earlier findings of a compact set of GPCRs in the *Ciona* genome. The high levels of sequence similarity to the human orthologs indicate that they serve functions that are general to vertebrate signaling biology and that *Ciona* can indeed be used as a model organism to study vertebrate GPCR signaling events. Extensive experimental characterization of the developmental regulatory network in *Ciona* points to the possibilities of similar studies for GPCR signaling pathways [45]. The clear one-to-one orthology between human and *Ciona* G protein repertoires that extends at the levels of protein family, subfamily and type, indicates a strong conservation in the evolution of the mechanistic basis of specificity and diversity of the chordate GPCR signaling pathways. Thus, the limited repertoire of heterotrimeric G protein complexes in *Ciona* provides an unique opportunity to extend the predictions to guide experimental studies to explore the basis of receptor activation, specificity and selectivity, phenotypic effects of GPCR signaling events, reinforce functional importance of existing receptor G protein/RGS protein interactions and for identification of new partners and downstream targets.

## Supporting Information

Data S1 Amino acid sequences of heterotrimeric G proteins and RGS proteins used as queries in BLAST and HMM search programs (FASTA format)
(0.09 MB TXT)

Data S2 Amino acid sequences of heterotrimeric G proteins and RGS proteins in *Ciona* (FASTA format)
(0.01 MB TXT)

Table S1 Accession numbers and classification of *Ciona* G protein and RGS protein sequences and human orthologs.
(0.05 MB DOC)

Table S2 Accession numbers for EST-hits of the *Ciona* heterotrimeric G proteins and RGS proteins in the Unigene database
(0.03 MB XLS)
