# Supplementary material for: The Repertoire of Heterotrimeric G Proteins and RGS Proteins in Ciona intestinalis
Source: PLoS One. 2009 Oct 6;4(10):e7349. doi: 10.1371/journal.pone.0007349 (PMC2752167; doi:10.1371/journal.pone.0007349)
Supplement: Table S1 — Accession numbers and classification of Ciona G protein and RGS protein sequences and human orthologs. (0.05 MB DOC) [file pone.0007349.s003.doc]

**Table S1**. Accession numbers and classification of *Ciona* G protein and RGS protein sequences and human orthologs.

| **Class** | **Family** | **Subfamily** | **Type** | **Human Sequence ID** | **Ciona Sequence ID (JGI- DB)** | **Chromosome locus**  **(Ciona)** | **GenBank Accession (Ciona)** |
| --- | --- | --- | --- | --- | --- | --- | --- |
| Gα | Gα_12/13_ | Gα_12_  Gα_13_  Unclassified Gα_12/13_ | Gα_12_  Gα_13_  Unclassified_Gα_12/13_ | Q03113  Q14344 | 287420 | chr_03p:233196-242732 | Not available |
|  | Gα_i/o_ | Gα gust  Gα_i_  Gα_o_  Gα_t_  Gα_z_ | Gα gust  Gα_i_  Gα_i1_  Gα_i2_  Gα_i3_  Gα_o_  Gα_o1_  Gα_t_  Gα_t1_  Gα_t2_  Gα_z_ | -  -  P63096 , O43383  Q96C71, P04899  P08754  Q8N6I9  P09471  Q16162  P11488  P19087  Q8N652, P19086 | 209567 | chr_01q:512951-526837 | BAB83918 |
|  | Gα_q/11_ | Gα_11_  Gα_14_  Gα_15/16_  Gα_q_  Unclassified Gα_q/11_ | Gα_11_  Gα_14_  Gα_15_  Gα_16_  Gα_q_  Unclassified Gα_q/11_ | P29992  O95837  P30679  P50148, Q6NT27 | 390373*,  272522 | scaffold_47:774800-775455  chr_03p:2004465-2012942 | XP_002127443 XP_002121062 |
|  | Gα_s_ | Gα_olf_  Gα_s_ | G_αolf_  Gα_s_ | Q86XU3, P38405  Q14455, P63092, Q5JWF2, O60726, O75632,O75633, Q14433 | 208025 | scaffold_145:148511-183627 | XP_002124602 |
|  | Other Gα | Other Gα | Other Gα |  | 220350,  273922*  202119,  287065 | chr_09p:3195649-3203859, chr_09p:3188147-3192040,  chr_04q:5667475-5668773,  chr_02q:5144690-5146848 | XP_002119438, XP_002119438  XP_002126618,  XP_002121575, |
|  | Unclassified Gα |  |  |  | 281048* | chr_08q:2585087-2588281 | Not available |
| Gβ | Gβ | Gβ | Gβ-1  Gβ-2  Gβ-3  Gβ-4  Gβ-5  Unclassified _Gβ | P62873  P62879  P16520, Q96B71  Q9HAV0  Q96F32,O14775  Q9UFT3 | 283613  297548 | chr_07q:149559-155532  chr_04q:5327824-5333559 | XP_002127296  XP_002126536 |
| Gγ | Gγ | Gγ | Gγ  Gγ-1  Gγ-2  Gγ-3  Gγ-4  Gγ-5  Gγ-7  Gγ-8  Gγ-10  Gγ-11  Gγ-12  Gγ-13  Gγ-e  Gγ-t1  Unclassified _Gγ | P59768  P63215  P50150  Q9Y3K8, P63218  O60262  [O14610, Q9UK08](http://www.uniprot.org/entry/O14610)  Q96BN9, P50151  P61952  Q9UBI6  Q9P2W3  P63211 | 225022, ci0100144696# | scaffold_36:73269-74918, scaffold_104:239245-241719 | Not available |
| RGS | RGS | RGS | RGS1(1R20)(BL34)  RGS2(G0S8)  RGS3  RGS4  RGS5  RGS6  RGS7  RGS8  RGS9  RGS10  RGS11  RGS12  RGS13  RGS14  RGS16(RGSR)  RGS17  RGS18  RGS19(GAIP)  RGS20 (RGSZ1)  RGS21  RGS22  Other RGS | [Q08116](http://www.uniprot.org/uniprot/Q08116)  [P41220](http://www.uniprot.org/uniprot/P41220)  [P49796](http://www.uniprot.org/uniprot/P49796)  [P49798](http://www.uniprot.org/uniprot/P49798)  [O15539](http://www.uniprot.org/uniprot/O15539)  [P49758](http://www.uniprot.org/uniprot/P49758)  [P49802](http://www.uniprot.org/uniprot/P49802)  [P57771](http://www.uniprot.org/uniprot/P57771)  [O54828](http://www.uniprot.org/uniprot/O54828)  [O43665](http://www.uniprot.org/uniprot/O43665)  [O94810](http://www.uniprot.org/uniprot/O94810)  [O14924](http://www.uniprot.org/uniprot/O14924)  [O14921](http://www.uniprot.org/uniprot/O14921)  [O43566](http://www.uniprot.org/uniprot/O43566)  [O15492](http://www.uniprot.org/uniprot/O15492)  [Q9UGC6](http://www.uniprot.org/uniprot/Q9UGC6)  Q9NS28  P49795  O76081  [Q2M5E4](http://www.uniprot.org/uniprot/Q2M5E4)  [Q8NE09](http://www.uniprot.org/uniprot/Q8NE09)  Other RGS | 383336*  288464  277300*  373767  266718*  270109*  276894*, 281930*,  219663  277281*,  277316*  267138*  276762*  ci0100153349# | chr_09p:2658531-2658882  chr_04q:741509-744033  chr_09p:691660-692363  chr_02q:5880833-5882566  chr_09p:2673066-2673836  chr_03q:4276107-4291112  chr_09p:1089032-1089868, chr_03q:2195340-2196071, chr_08q:6763092-6773632  chr_09p:2667269-2668007,  chr_09p:2680448-2681371  chr_01q:3779313-3780773  chr_09p:514469-515397  Scaffold_224:121853-122488 | XP_002122056  XP_002130938  XP_002126381  XP_002132032  XP_002122409  XP_002125747  XP_002124469, XP_002121872,  Not available  XP_002121879, XP_002122539  XP_002123933  Not available  NP_001071660. |

* These sequences were fragments in JGI *Ciona intestinalis* genome database Ver2.0 and manually edited

# These sequences have accession numbers corresponding to Ciona genome database Ver 1.0
